# Autonomous Energy Harvester Based on Textile-Based Enzymatic Biofuel Cell for On-Demand Usage †

**DOI:** 10.3390/s20175009

**Published:** 2020-09-03

**Authors:** Seonho Seok, Cong Wang, Elie Lefeuvre, Jungyul Park

**Affiliations:** 1 Center for Nanoscience and Nanotechnology (C2N), University-Paris-Saclay, 91120 Palaiseau, France; elie.lefeuvre@universite-paris-saclay.fr; 2 Department of Mechanical Engineering, Sogang University, Mapo-gu, Seoul 04107, Korea; wang.tsung@gmail.com

**Keywords:** textile-based glucose fuel cell, energy harvesting, DC–DC converter, power management

## Abstract

This paper presents an autonomous energy harvester based on a textile-based enzymatic biofuel cell, enabling an efficient power management and on-demand usage. The proposed biofuel cell works by an enzymatic reaction with glucose in sweat absorbed by the specially designed textile for sustainable and efficient energy harvesting. The output power of the textile-based biofuel cell has been optimized by changing electrode size and stacking electrodes and corresponding fluidic channels suitable for following power management circuit. The output power level of single electrode is estimated less than 0.5 μW and thus a two-staged power management circuit using intermediate supercapacitor has been presented. As a solution to produce a higher power level, multiple stacks of biofuel cell electrodes have been proposed and thus the textile-based biofuel cell employing serially connected 5 stacks produces a maximal power of 13 μW with an output voltage of 0.88 V when load resistance is 40 kΩ. A buck-boost converter employing a crystal oscillator directly triggered by DC output voltage of the biofuel cell makes it possible to obtain output voltage of the DC–DC converter is 6.75 V. The efficiency of the DC–DC converter is estimated as approximately 50% when the output power of the biofuel cell is tens microwatts. In addition, LT-spice modeling and simulation has been presented to estimate power consumption of each element of the proposed DC–DC converter circuit and the predicted output voltage has good agreement with measurement result.

## 1. Introduction

**Figure 1:**
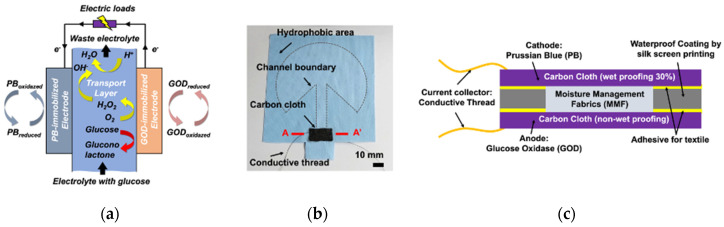
A single electrode biofuel cell: (**a**) working principle (**b**) fabricated biofuel cell (**c**) cross-section of AA’ of the electrodes.

Wearable devices have gained significant interest from academia and industry owing to their immense applications such as biomedical, health and entertainment, etc. [1,2]. To realize the internet of things (IoT) for human body data or human–machine interactions, wearable devices need to be capable of undertaking multiple complex tasks and require long term and sustainable energy sources. Concerning the energy sources for wearable electronics, flexible and stretchable batteries have attracted attention due to their suitable nature for wearable devices [3,4]. Stacked thin-film planar structure batteries can be integrated into compliant substrate such as a paper [3]. This approach has evolved into the shape of a fiber or wire allowing integration with wearable garments [5,6,7,8,9]. Recently, nanogenerators based on different principles such as piezoelectric, light, triboelectric, and thermoelectric have been reported [10,11,12,13,14,15,16,17]. Besides, stretchable batteries have been proposed using bridge-island design, winding fiber, stretchable fabric, etc. [18,19,20,21]. As an alternative solution, wearable energy harvesters harnessing energy directly from the wearer’s body based on biofuel cell have been recently reported [22]. Biofuel cells (BFCs) rely on the use of biocatalytic redox enzymes to convert chemical energy into electrical energy. Most of the reported biofuel cells use blood glucose as a fuel and thus complex invasive implantation within the wearer is indispensable [23,24,25]. Therefore, the need for easy-to-wear non-invasive BFCs as a wearable energy source is highly increased. To address the above issues, the wearable power supply needs to be developed, which enables to generate electric power from body fluid for sustainable operation [26,27]. Many biofuel cells used textile and paper substrates as flexible electrodes or fuel reservoirs [28,29,30], and the previously reported paper-based biofuel cells had a maximum power density of 13.5 μW/cm^2^ in Zhang et al. [31], 5.5 μW/cm^2^ in Fraiwan et al. [32], etc. However, due to biofuel depletion and solvent evaporation in the hydrostatic electrolyte, the output power significantly decreased and the lifetime was very limited; electrolyte refilling or paper exchange was needed. Some studies used a paper substrate as a fuel supplier to improve the output power by the induced capillary flow [33,34,35,36]. However, studies on the critical role of high-speed, sustainable capillary flow generation have rarely been carried out, and there is still lots of room for improvement in terms of the durability and lifetime of the biofuel cells. The capillary-driven flow not only supplies the fuel for the redox reaction, but also enables efficient mass transfer (e.g., oxygen or intermediate products) between the anode and cathode, which mainly depends on flow speed. Therefore, a wearable textile-based glucose fuel cell has been proposed using moisture management fabrics (MMF) for improved and long-term power generation as shown in Figure 1 [37] © 2019 IEEE. MMF is widely used as basic materials for a sportswear and shows very fast sweat absorption and water evaporation. It is composed of polyester with modified cross-sectional shapes for quick water absorbing and wicking and has the series of closely spaced channels for high evaporation. The balance between fast water evaporation and absorption enables high flow rate and continuous flow within MMF. Owing to low-cost and scalable fabrication process, it can be easily integrated into clothes, socks or underwear to utilize the human body fluid like sweat, tear or urine as fuel resource to generate energy for wearable devices. Concerning power management circuit for the energy harvester, it typically consists of rectifier, DC–DC converter, and power storage element. Since power storage element usually requires a DC voltage input, an energy harvesting circuit needs AC–DC rectifier after the energy harvester like in piezoelectric energy harvesters and electromagnetic power devices [38,39,40]. In case of BFCs, they provide DC output current enabling direct powering of power storage elements. However, direct powering wearable devices with BFCs may be undesirable due to its low output voltage and load dependent characteristics [41]. Thus, a DC–DC converter is required as an interface circuit between biofuel cell and wearable electronics. The role of power management circuit for energy harvesters is for delivering maximum power from energy harvesters to energy storage element, for example, battery. Basically, it is achieved by matching load resistance with the internal resistance (or impedance) of the energy harvesters. For dealing with low input power or voltage of energy harvesters based on biofuel cells, intermediate capacitor for accumulation of enough charge to transfer to output [42] or micro-transformer for step-up oscillator [43] have been used. Alternatively, parallel multiple fuel cell elements are proposed for most efficient energy harvesting [44]. As biofuel cells typically produce non-constant output voltage, feedforward control of DC–DC PWM (Pulse Width Modulation) boost converter is a way to achieve high efficiency power conversion significantly improving the performance of this DC–DC converter. In this paper, autonomous textile-based enzymatic biofuel cell combined with DC output voltage-driven DC–DC converter is presented. The DC output voltage of biofuel cell is optimized by studying the dependence of output power on number and size of the biofuel cell’s electrode. Brief introduction of textile-based enzymatic biofuel cell is presented in Section 2. Section 3 discusses electrical characterization of the textile-based enzymatic biofuel cell. Power management circuit simulation and experimental results are presented in Section 4. Finally, Section 5 summarize our works.

## 2. Textile-Based Enzymatic Biofuel Cell

The principle of the fabricated textile-based enzymatic biofuel cell is depicted in Figure 1, which has been previously reported by Wang et al. [45] The textile-based glucose fuel cell consists of MMF, carbon cloth-based cathode and anode, and conductive thread as shown Figure 1a. As depicted in Figure 1b, the cathode and anode were fabricated by coating carbon cloth with Prussian blue (PB) nanoparticles and glucose oxidase (GOD), respectively. Figure 1c shows cross-section of the electrode of the biofuel cell. In the presence of glucose, the enzyme-catalyzed reaction occurs between glucose and the oxidized form of GOD at the anode. The dissolved oxygen in the aqueous solution as a natural mediator could be spontaneously reduced to hydrogen peroxide by the electrocatalytic reaction of the reduced form of GOD [46,47,48]. Hydrogen peroxide produced by the GOD-modified anode reacts with the reduced form of PB at the cathode and can be finally reduced to neutral water by the following reactions:

(1) $$GOD\left(FADH_{2}\right)↔GOD\left(FAD\right)+2H^{+}+2e^{-}$$

(2) $$GOD\left(FAD\right)+glucose\to GOD\left(FADH_{2}\right)+gluconolactone$$

(3) $$GOD\left(FADH_{2}\right)+O_{2}\to GOD\left(FAD\right)+H_{2}O_{2}$$

(4) $$H_{2}O_{2}\to O_{2}+2H^{+}+2e^{-}$$

(5) $$K_{4}Fe_{4}^{II}{\left[Fe^{II}{\left(CN\right)}_{6}\right]}_{3}+2H_{2}O_{2}\to Fe_{4}^{III}{\left[Fe^{II}{\left(CN\right)}_{6}\right]}_{3}+4K^{+}+4OH^{-}$$

(6) $$Fe_{4}^{III}{\left[Fe^{II}{\left(CN\right)}_{6}\right]}_{3}+4K^{+}+4e^{-}↔K_{4}Fe_{4}^{II}{\left[Fe^{II}{\left(CN\right)}_{6}\right]}_{3}$$

Especially, the carbon cloth for the cathode is treated with 30% PTFE to enable the air exchange within the cathode. Hence, the PB could be electrochemically reduced again enabling spontaneous recharging and long-term use [49,50]. MMF in the middle functions as transport layer, on which fluid channels are patterned with hydrophobic boundary, for fuel resource absorption and transport. Owing to the continuous and high-speed flow in the MMF, the proposed textile-based biofuel cell is able to work for more than 12 h (Figure S1). The conductive thread is used as a current collector to connect with the external loads. 

The fabrication process of the biofuel cell is the same as that described in our previous studies in detail [45] (Figure S2). Carbon fabric without waterproofing and with 30% polytetrafluoroethylene waterproofing (AvCarb^TM^ 1071 HCB, Ballard Material Products) was used for the substrate of the anode and cathode, respectively. Commercial GOD (from *Aspergillus niger*, EC 1.1.3.4, Type X-S, Sigma-Aldrich, Seoul, Korea) immobilization on the carbon fabric (1 cm^2^) was conducted at 4 °C with 0.1 M acetate buffer solution (mixed solution of acetic acid and sodium acetate) at pH 5 and GOD concentrations (6 mg/mL) over 6 h, followed by 15-min washing with acetate buffer solution to remove any residue. The GOD-immobilized carbon fabric was then dried and stored in a fridge at 4 °C before use [51]. A mass of 6 mg PB nanoparticles, 2 mg carboxylated multi-walled carbon nanotube (MWCNT), and 5 mL of 5 wt% Nafion was sequentially added to 100 mL isopropyl alcohol and 30 mL DI water, followed by vortex-mixed and sonicated to prepare 1 cm^2^ of PB-immobilized cathode [52]. The suspension was brushed onto the carbon fabric, layer-by-layer, and the as-prepared cathode was baked overnight at 100 °C to stabilize the PB/MWCNT composite. MMF is single jersey knitted fabrics composed of 92% polyester and 8% polyurethane. The microscope image of MMF and the SEM images of the GOD- and PB-immobilized carbon cloth are shown in Figure S3. To guide the fuel source to the electrode region, 10-mm-wide channels were patterned onto the transport layers with hydrophobic boundaries using a silk screen printing procedure [53]. Before attaching the carbon fabrics-based electrodes onto the transport layer, a stainless steel conductive thread (Adafruit 640, New York City, NY, USA) was hand-sewn onto the carbon fabric. Finally, the two pieces of carbon fabric with the cathode and anode were bonded to the top and the bottom side of the transport layer, respectively, with solvent-free fabric adhesive (UHU textile).

**Figure 2:**
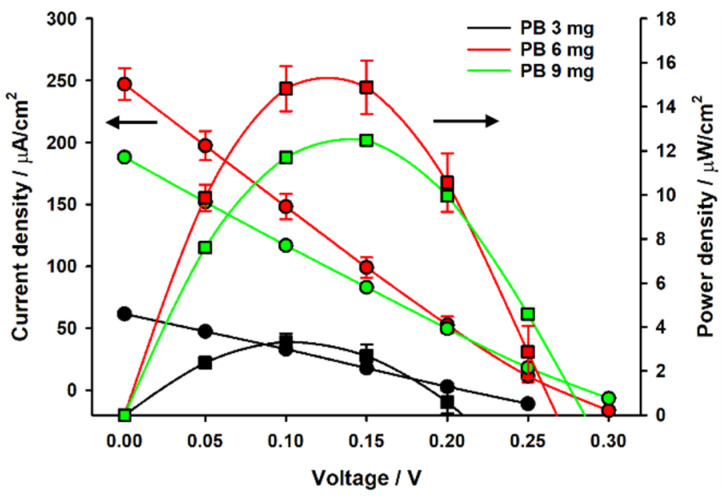
Static electrical characteristics of single biofuel cell.

**Figure 3:**
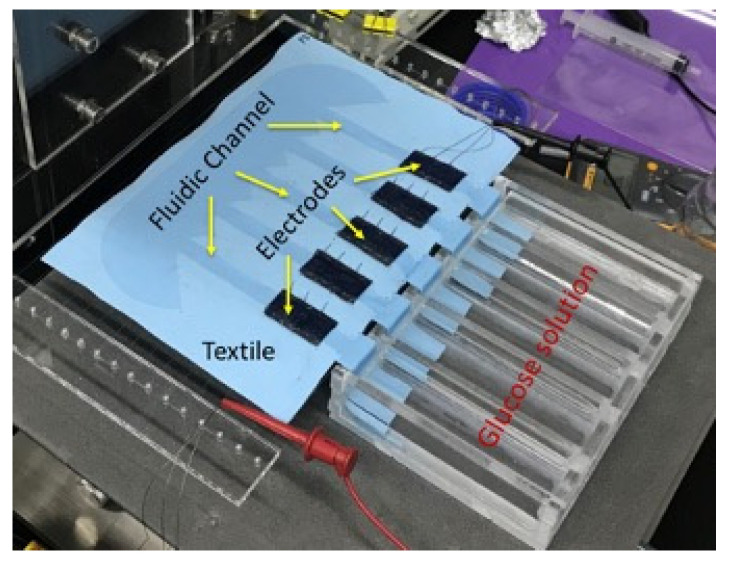
Biofuel cell with serially connected 5 stacks and modified fluidic channel.

**Figure 4:**
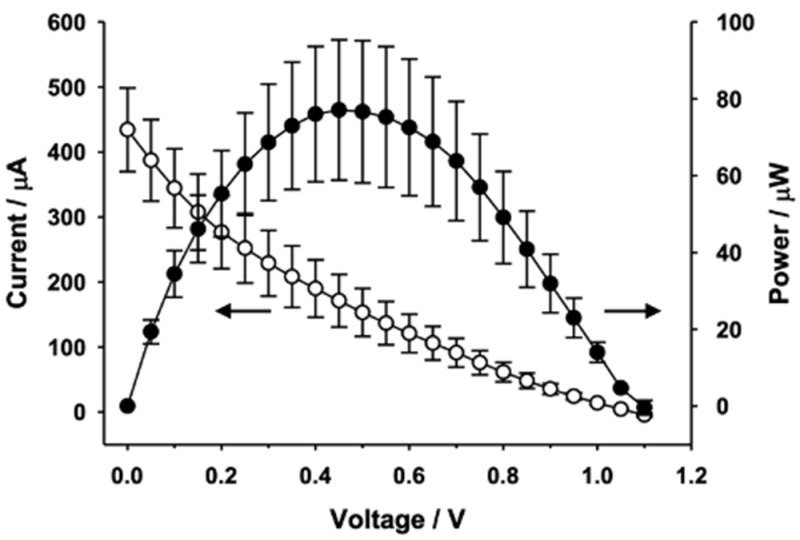
Static electrical characteristics of biofuel cell with serially connected 5 stacks and modified fluidic channel.

Voltage-Current response of the fabricated biofuel cell has been measured in the following way; D-(+)-glucose (G8270, Sigma Aldrich) solutions were prepared in phosphate-buffered saline (PBS, pH 7.4), subjected to mutarotation at room temperature for 24 h, and stored at 4 °C before use. After the glucose solution was added to the biofuel cell, the voltage and current signals were recorded using the LabVIEW program (National Instruments) by a computer which was connected to a picoammeter/voltage source (6487, Keithley Instrument) through a general-purpose interface bus card (PCI-GPIB, National Instruments). The current–voltage (I–V) curves were obtained in a potentiostatic mode, and each voltage step was maintained for 10 s to acquire steady-state I–V curves. Figure 2 shows measurement results of I-V curve of the single electrode biofuel cell. The biofuel cell device with single electrode provides output power of 15 μW/cm^2^ with V_oc_ = 0.3 V and I_sc_ = 200 μA, where V_oc_ is open circuit voltage and I_sc_ is short circuit current. Through electrical characterization, it is found that maximal power transfer is realized when V_out_ = V_oc_/2 and I_out_ = I_sc_/2. Thus, output voltage of the biofuel cell with single electrode at maximal power output mode is theoretically 0.15 V since open circuit voltage (V_oc_) is 0.3 V. The output voltage amplitude becomes important when the selected oscillator component of power management circuit needs at least 1 V threshold voltage for triggering clock signal as will be explained later. Therefore, it is required to increase the DC output voltage of the biofuel cell for autonomous operation of power management circuit. It has been achieved through series connection of 5 stacks of electrodes and modified fluidic channel and reservoir as shown in Figure 3. The 5 electrode-stacked biofuel cell provides 70 μW of maximal power when V_out_ = 0.5 V and I_out_ = 140 μA, as presented in Figure 4. It should be noted that output current and output power levels have substantial fluctuation as represented by error bars in Figure 4.

## 3. Electrical Characterization of Biofuel Cell with Load Resistance

**Figure 5:**
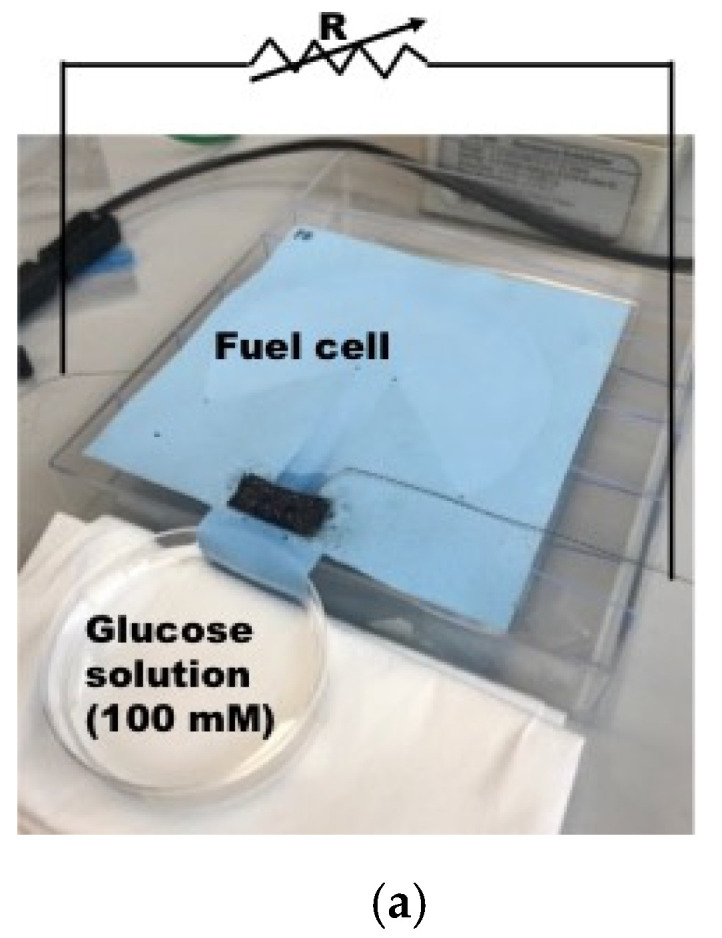

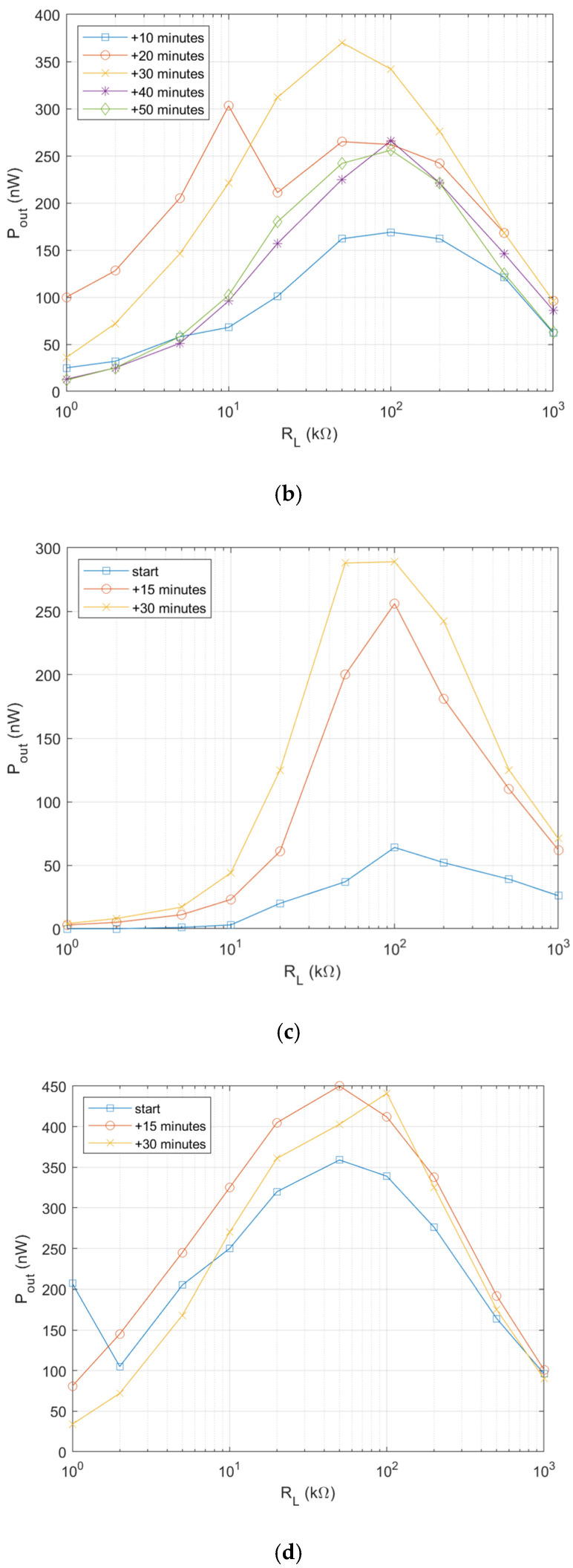
Characterization of output power of single electrode biofuel cell. (**a**) Output power measurement with load resistance method [37] © 2019 IEEE; (**b**) Output power measurement result; sample 1; (**c**) Output power measurement result; sample 2; (**d**) Output power measurement result; sample 3.

The objective of electrical characterization of the fabricated biofuel cell is to find internal resistance for the design of optimal power management circuit design. The biofuel cells fabricated in the previous section has been characterized as a function of load resistance, while a glucose solution of 100 mM is fed from one end of the textile-based biofuel cell as depicted in Figure 5a. Output power evolution of 3 biofuel cell samples from same fabrication group has been presented in Figure 5b–d. The output power of the biofuel cell starts to increase as feeding time of glucose solution is increased. However, it tends to be reduced after 30 min as seen in Figure 5b. The optimal load resistance for maximum power transfer is found around 100 kΩ for all of the samples and maximum power obtained ranges from 300 nW to 450 nW with the electrode (black colored patch in the figure) size of 1 cm^2^. Output voltages are measured less than 200 mV which is too low to trigger X-tal oscillator component for DC–DC converter for autonomous operation as explained earlier.

**Figure 6:**
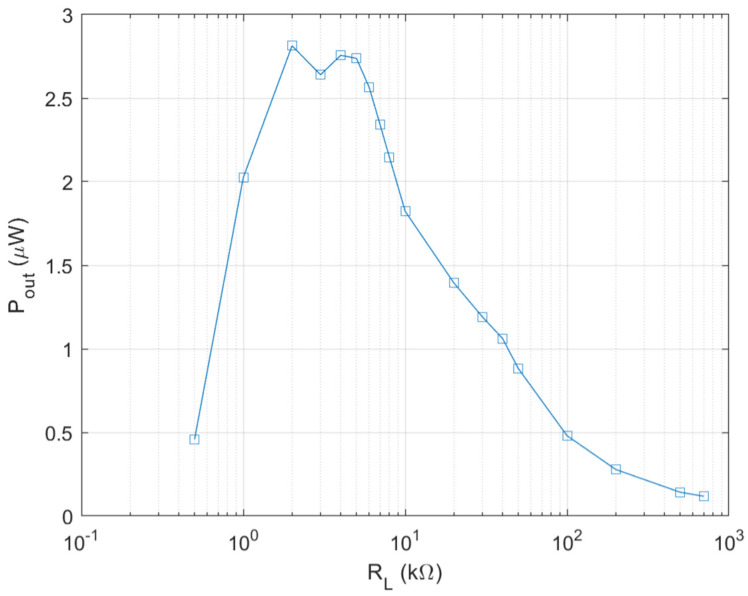
Output power of enlarged single electrode biofuel cell.

Therefore, it is necessary to enhance output power and output voltage of the biofuel cell for subsequent DC–DC converter to be operated in an autonomous mode. Firstly, this is done by increasing the electrode size by 1.5 times of the initial design and a wind of 0.8 m/s is applied to the biofuel cell with a fan. The wind plays a role of accelerating of liquid evaporation and thus preventing the channel from being saturated by the glucose solution. The wind speed corresponds to the slow walking speed of humans, which is about 1.4 m/s [18]. The output power measurement result of the biofuel cell with the enlarged electrode is shown in Figure 6. As expected, the biofuel cell with 1.5 times larger electrode shows output power density of 1.4 µW/cm^2^. The optimal load resistance is found to be 2–4 kΩ. For comparison, the initial biofuel cell provides output power density of 0.3–0.44 µW/cm^2^ with optimal load resistance of 100 kΩ. It is found that the output voltage is around 0.3 V even if output power level is substantially enhanced. 

**Figure 7:**
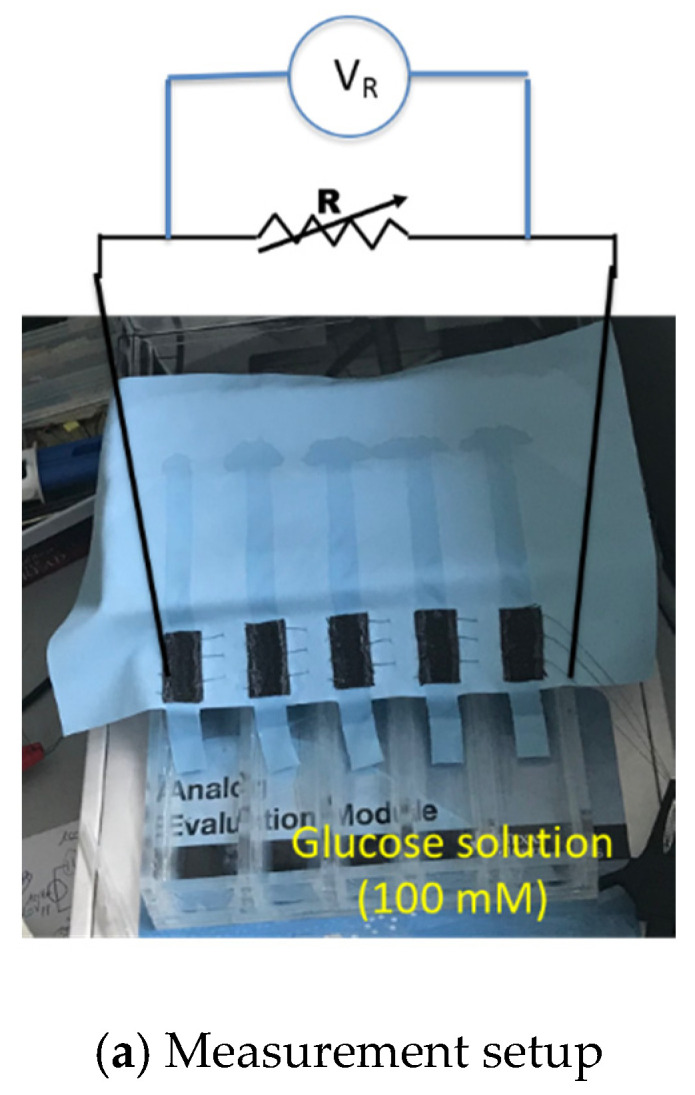

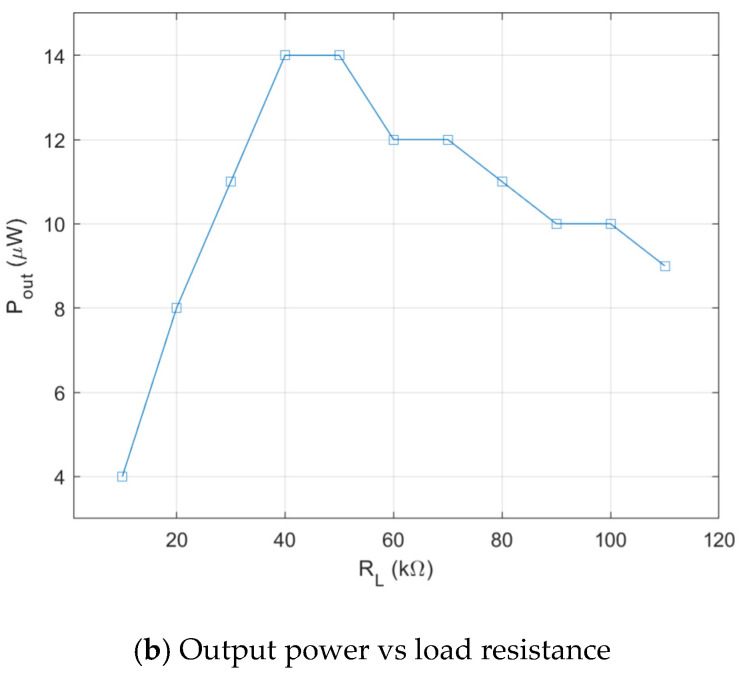
Characterization of output power of serially connected 5-stack biofuel cell.

As explained in previous section, the biofuel cell is modified to get a serially connected 5-stack electrode to increase its output DC voltage. Figure 7a shows output power measurement setup with resistance load. The glucose solution is fed through 5 separate MMF fabric from the reservoir. Output power measurement result of the biofuel cell with 5 stacks is shown in Figure 7b. The maximum output power increases up to 14 μW when load resistance is 40–60 kΩ. The output voltage of the biofuel cell with 5 stacks of electrode is 0.84 V, which is evidently high enough to trigger the X-tal oscillator of DC–DC converter.

## 4. Power Management Circuit

**Figure 8:**
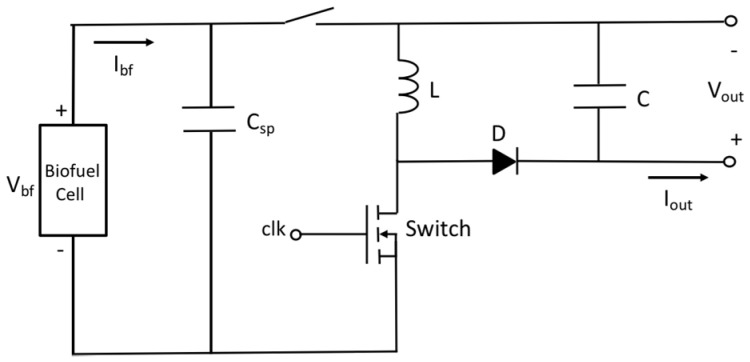
Schematic of two-staged power management circuit for single electrode biofuel cell.

**Figure 9:**
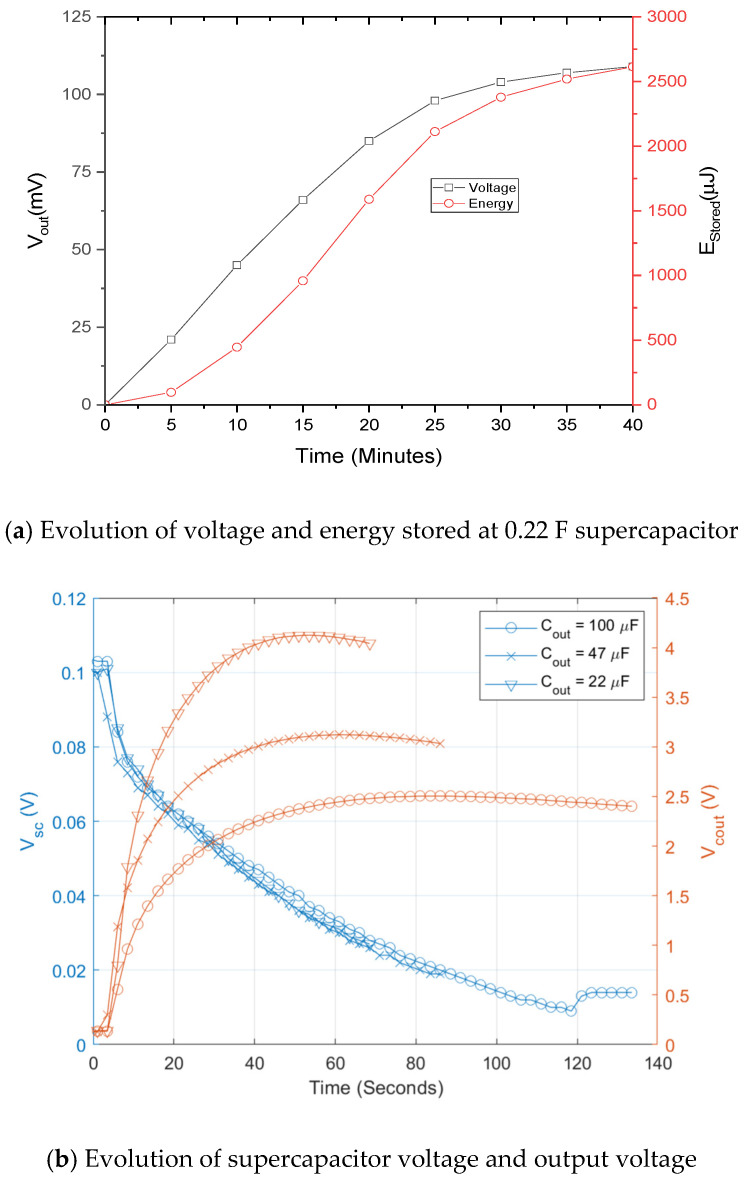
Measurement results of two-staged power management circuit for single electrode biofuel cell [37] © 2019 IEEE.

It has been estimated with load resistance method that the fabricated biofuel cells have output power level of 300–450 nW with single electrode and that of 13 μW with 5 stacks of electrodes in the previous section. For the single electrode biofuel cell, two stages approach including charging and DC–DC conversion has been adopted to compensate the low output voltage and low output power. Figure 8 shows schematic of the two-staged power management circuit. It is operated as follows: (1) small output current I_bf_ is charged in a supercapacitor C_sp_ of 0.22 F to get enough voltage for power conversion of DC–DC converter; (2) By switching on after superconductor charging, DC–DC converter is then connected to supercapacitor for step-up of the voltage output. According to Figure 9a, DC voltage conversion starts after supercapacitor charging time of 40 min when the input voltage of DC–DC converter becomes 100 mV. The DC–DC converter is used to step up the supercapacitor voltage suitable for conventional battery charging. Energy stored at the supercapacitor is converted through inductor L and diode D before it is charged into output capacitor C by adjusting duty cycle of clock control signal of switch. Evolution of the voltage on supercapacitor and C at different conditions is presented in Figure 9b. Output voltage is ranged from 2.5 V to 4 V and conversion time is around one minute for all the cases.

**Figure 10:**
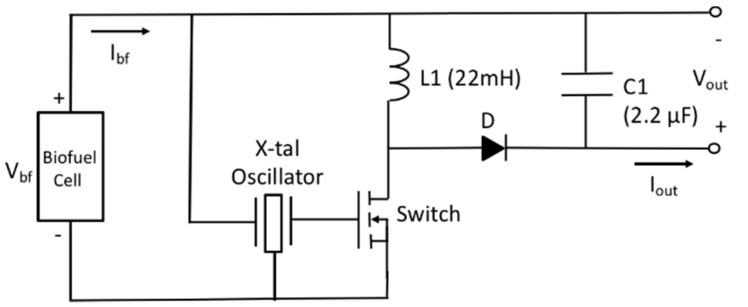
Schematic of biofuel cell energy harvester with power management circuit.

As the biofuel cell with 5 stacks provides higher output voltage as well as higher output power than that of the single electrode biofuel cell, the two-stage power management circuit can be replaced with a simple circuit. Our approach is to use the DC output voltage of the biofuel cell as a trigger signal of commercial quartz oscillator (OV-7604-C7, low power crystal oscillator 32.768 kHz, Micro Crystal Switzerland). Referring to datasheet of the quartz oscillator, required minimum DC voltage is 1.2 V. This minimal DC voltage could be made from output of biofuel cell by using a transformer [43]. In our case, constraint on device size is not a concern as it is based on textile for workout wear and thus multiple stacks of current-generating electrode having corresponding fluidic channel and common reservoir at the end of the channel have been used to create wanted DC output voltage. A schematic of the biofuel cell energy harvester with power management circuit is shown in Figure 10. The X-tal oscillator is used as clock generator for switch element of DC–DC converter. Frequency and duty cycle of the switching action determines input impedance of the power management circuit given in Equation (7).

(7) $$R_{in}=\frac{2Lf_{sw}}{D^{2}}$$

**Figure 11:**
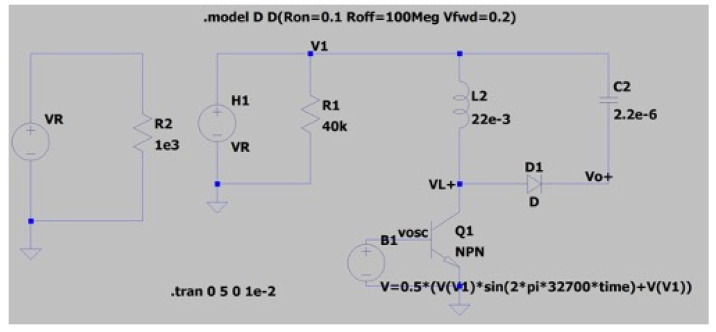
LT-spice circuit model of biofuel cell with power management circuit.

**Figure 12:**
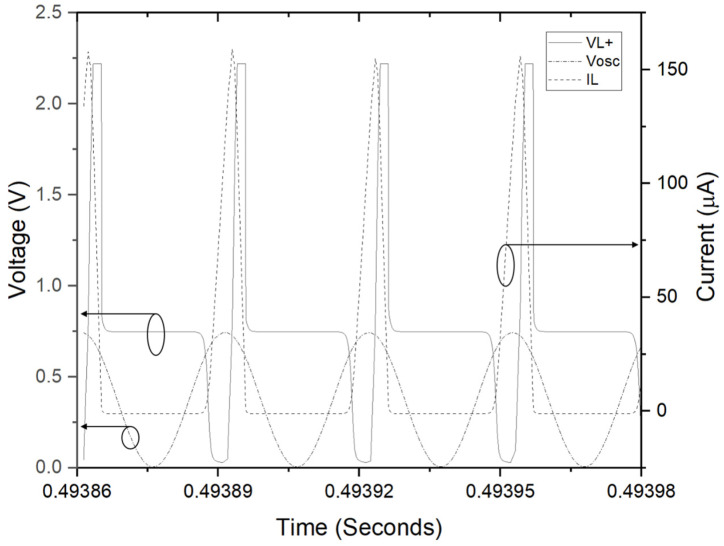
Simulation results; inductor current (IL), oscillator voltage (Vosc), and inductor voltage (VL+).

As frequency and duty cycle of the X-tal oscillator is fixed 32.7 kHz and 50% respectively, input impedance is expected to be 57.6 kΩ. The operating point of power management circuit is in quasi-optimal range for power transfer. Next, the LT-spice circuit model of the biofuel cell with power management circuit, presented in Figure 11, has been simulated to find theoretical efficiency of the power management circuit. The biofuel cell is modelled as current-controlled voltage; the experimental results of maximum power transferred to load resistance have been used to determine current source value and gain of the dependent voltage. Concerning clock signal for transistor switch, it is defined as behavior source referring to DC output voltage, V1. A sinusoidal source with frequency of 32.7 kHz is used for the experiment. Figure 12 shows waveforms of inductor current (I_L_), oscillator voltage (V_osc_), and inductor voltage (V_L+_). It shows typical behavior of DC–DC converter and the rms values of important elements for power consumption of the DC–DC converter are 50.4 μA for I_L_, 44.4 μA for I_c_(Q1). The theoretical efficiency of the power management circuit based on the simulation results has been estimated and summarized in Table 1. Most of the input power from biofuel cell is dissipated for switching operation and the estimated efficiency of the power management circuit is 74.5%. Note that resistance of MOS and inductor elements are assumed to get largest value from commercial products and an ideal diode element is considered.

**Figure 13:**
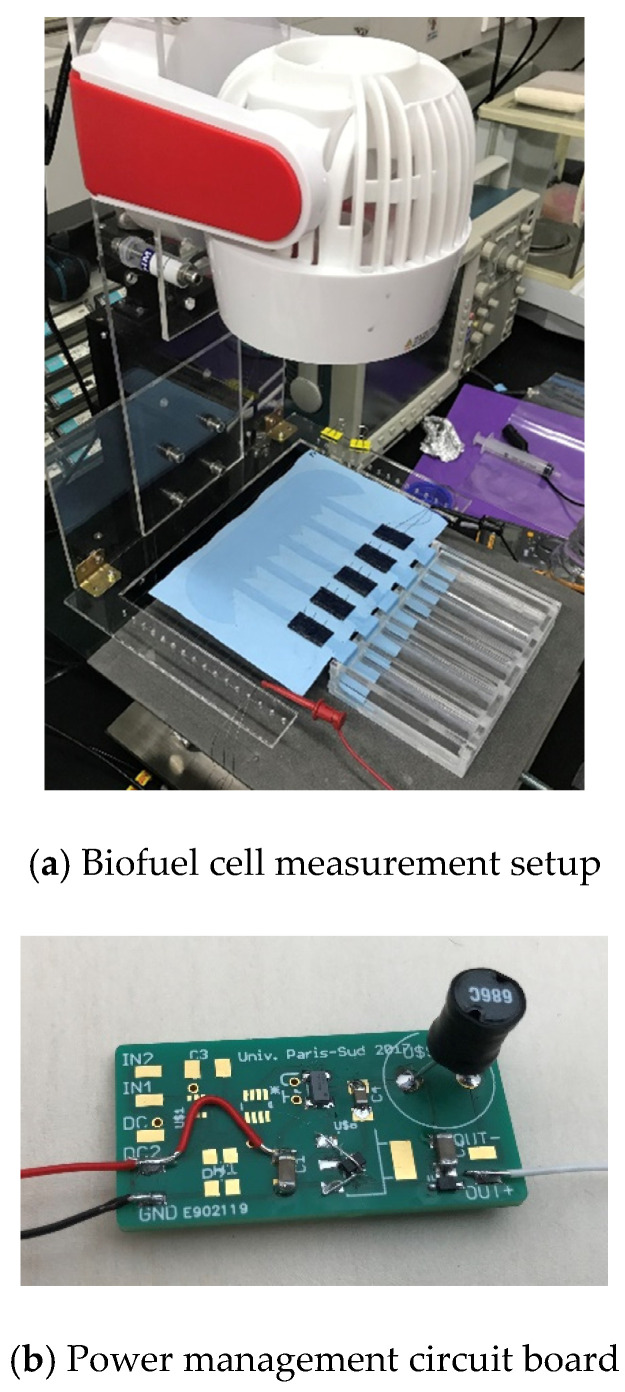
Measurement setup and power management board for biofuel cell with 5 electrodes.

The biofuel cell with 5 electrodes is measured with a power management circuit board, as shown in Figure 13. During characterization, a wind of 0.8 m/s is applied onto the biofuel cell. The wind plays the role of accelerating liquid evaporation and thus preventing the channel from being saturated by the glucose solution. 

**Figure 14:**
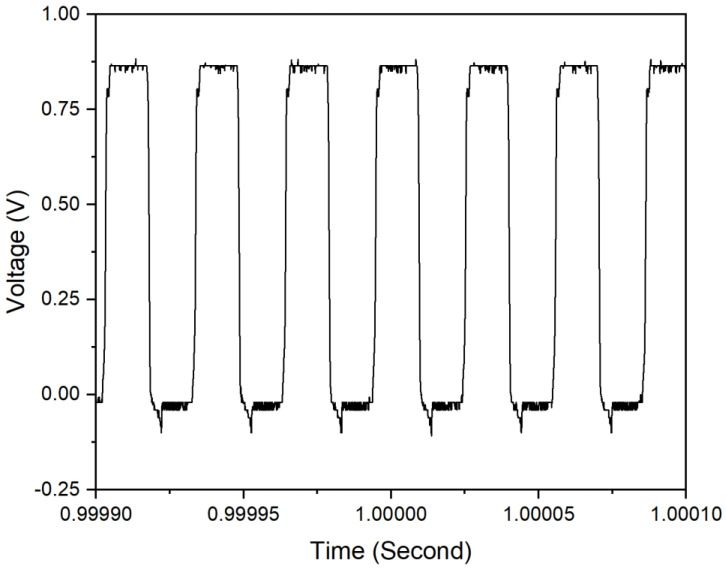
Output signal of oscillator.

The output signal of the oscillator is first checked, and it has 0.9 V peak amplitude of 32.7 kHz oscillation frequency as presented in Figure 14. 

**Figure 15:**
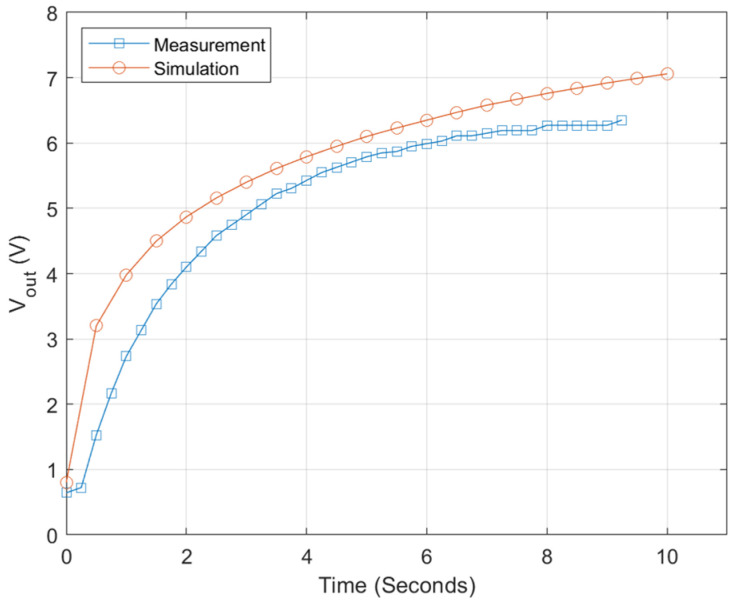
Output voltage of power management circuit.

**Figure 16:**
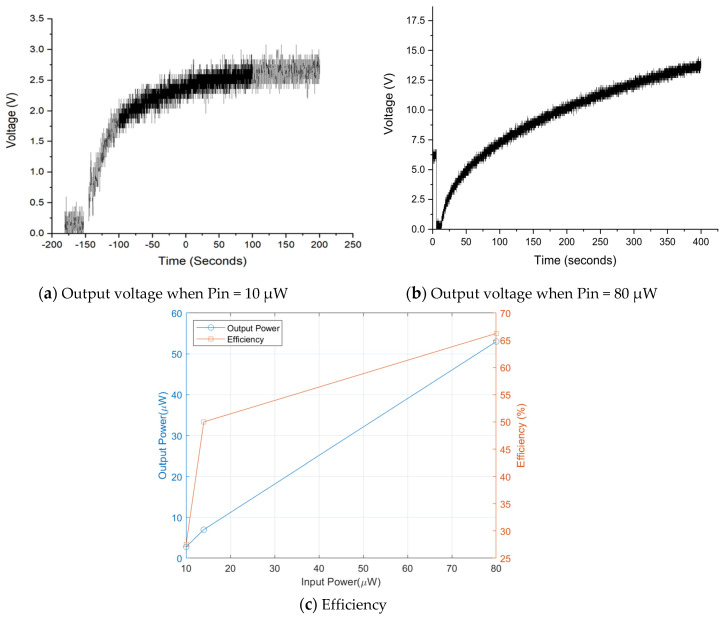
Efficiency of power management circuit as function of input power.

The output voltage of the power management circuit has been measured, and it showed typical capacitor charging characteristics as presented in Figure 15. Output power is estimated to be 6.9 μW and thus measured efficiency is around 50%. Disparity between theoretical and experimental is caused by power wastes through measurement environment and diode resistance etc. Figure 16a,b shows output voltage charging 220 μF capacitance at different input powers. The efficiency of the DC–DC converter is then measured as a function of input power as shown in Figure 16c. The efficiency of the power management circuit reaches 70% with input power of 80 μW, while it falls to 30% at input power of 10 μW. It might come from the trigger signal quality taken from biofuel cell output for the X-tal oscillator. Note that actual minimal voltage for the X-tal oscillator is found 0.7 V because the oscillator has been turned off at lower trigger voltage. 

Even though the glucose concentration for this work (100 mM) is higher than that in human sweat (about 0.1 mM), the possibility of autonomous DC-to-DC covert from our proposed power management system was successfully validated when the electricity is harvested from the textile-based enzymatic biofuel cell. We obtained some experimental results to investigate the effect of glucose concentration on power generation in a single biofuel cell (Figure S4) and found that the generated OCV is similar with respect to the variation of glucose concentration. Therefore, the oscillator would still work out with the 5 electrode-stacked biofuel cells because the out voltage exceeds the start-up voltage in the proposed circuits. Obviously, the energy conversion efficiency would be reduced due to a low power density in the low concentration. Therefore, the ASIC circuit having the same function of the proposed system is needed to work out in real human sweat.

## 5. Conclusions

An energy harvester based on textile-based enzymatic biofuel cell has been presented for wearable electronics. A textile-based biofuel cell using human body fluid is very useful in that it is an easy-to-wear, non-invasive way to achieve a wearable energy source. The output power level of the single electrode sizing of 1 cm^2^ is measured at about several hundred nanowatts, while a 5-stack biofuel cell produces tens of microwatts. It is also found that wind plays an important role to get higher and time-stable output power level. A two-staged power management circuit with charging supercapacitor has been implemented for submicrowatt-level biofuel cell, whereas X-tal oscillator-driven buck-boost DC–DC converter is used for a 5-stack biofuel cell due to its high voltage output, allowing to trigger X-tal oscillator. The 5-stack biofuel cell achieves a minimum voltage of 0.7 V for X-tal osillator triggering, which attains an efficiency of 50% when input power is tens of microwatts. Besides, power consumption of the proposed DC–DC converter circuit has been estimated through LT-spice modeling and simulation. The output voltage of the biofuel cell has shown time-dependent fluctuation even with the same load resistance. Hence, it is necessary to comply such a fluctuation to achieve higher efficiency power management circuit. As input resistance depends on duty cycle of switch control signal, a PWM (Pulse Width Modulation) enabling adaptive duty cycle will be adopted into the power management circuit for the biofuel cell in near future. Furthermore, ASIC circuit and its 3D packaging and integration technique should be developed due to the flexible nature of the wearable devices to integrate the biofuel cell and related power management circuit with wearable devices and sensors. 

## Figures and Tables

**Table 1:** Estimated power consumption of the power management circuit.

Items	Governing Equations	Values
Output power of biofuel cell P_bf_	V_bf_ × I_bf_ = 0.88 V × 15.9 μA	14 μW
Oscillator power P_osc_	0.9 V × 300 nA	0.27 μW
Switching power P_sw_	C_gate_ × V_sw_^2^ × f_sw_ = 100 pF × (0.9 V)^2^ × 32.7 kHz	2.65 μW
MOS power P_MOS_	Ic(Q1)_rms_^2^ × R_on,MOS_ = (111 μA)^2^ × 1 Ω	12.3 nW
Inductor power P_ind_	IL_rms_^2^ × R_ind_ = (119 μA)^2^ × 45 Ω	0.64 μW
Efficiency P_out_/P_in_	(P_bf_ − P_osc_ − P_sw_ − P_MOS_ − P_ind_)/P_bf_	74.5%

## Supplementary Materials

The following are available online at https://www.mdpi.com/1424-8220/20/17/5009/s1, Figure S1: Fabrication process of the textile-based enzymatic biofuel cell. Figure S2: Long-term output of the biofuel cells when connected with an external resistance (1200 Ω). Figure S3: (a) The microscope image of MMF and the SEM images of (b) the GOD- and (c) PB-immobilized carbon cloth. Figure S4: Variation of power generation according to the glucose concentration (a) The current density and (b) the maximum power density.

## References

[1] Ghomian T., Mehraeen S. (2019). Survey of energy scavenging for wearable and implantable devices. Energy.

[2] Yi F., Ren H., Shan J., Sun X., Wei D., Liu Z. (2018). Wearable energy sources based on 2D materials. Chem. Soc. Rev..

[3] Hu L., Wu H., la Mantia F., Yang Y., Cui Y. (2010). Thin, flexible secondary Li-ion paper batteries. ACS Nano.

[4] Gaikwad A.M., Whiting G.L., Steingart D.A., Arias A.C. (2011). Highly flexible, printed alkaline batterie based on mesh-embedded electrodes. Adv. Mater..

[5] Park J., Park M., Nam G., Lee J.-S., Cho J. (2015). All-solid-state cable-type flexible zinc–air battery. Adv. Mater..

[6] Weng W., Sun Q., Zhang Y., Lin H., Ren J., Lu X., Wang M., Peng H. (2014). Winding aligned carbon nanotube composite yarns into coaxial fiber full batteries with high performances. Nano Lett..

[7] Kwon Y.H., Woo S.-W., Jung H.-R., Yu H.K., Kim K., Oh B.H., Ahn S., Lee S.-Y., Song S.-W., Cho J. (2012). Cable-type flexible lithium ion battery based on hollow multi-helix electrodes. Adv. Mater..

[8] Qu H., Semenikhin O., Skorobogatiy M. (2014). Flexible fiber batteries for applications in smart textiles. Smart Mater. Struct..

[9] Liu T., Liu Q.-C., Xu J.-J., Zhang X.-B. (2016). Cable-type water-survivable flexible Li-O_2_ battery. Small.

[10] Wang Z.L., Song J. (2006). Piezoelectric nanogenerators based on zinc oxide nanowire arrays. Science.

[11] Qin Y., Wang X., Wang Z.L. (2008). Microfibre–nanowire hybrid structure for energy scavenging. Nature.

[12] Kotipalli V., Gong Z., Pathak P., Zhang T., He Y., Yadav S., Que L. (2010). Light and thermal energy cell based on carbon nanotube films. Appl. Phys. Lett..

[13] Fan F.R., Tian Z.Q., Wang Z.L. (2012). Flexible triboelectric generator. Nano Energy.

[14] Gong Z., He Y., Tseng Y.H., O’Neal C., Que L. (2012). A micromachined carbon nanotube film cantilever-based energy cell. Nanotechnology.

[15] Mao Y., Geng D., Liang E., Wang X. (2015). Single-electrode triboelectric nanogenerator for scavenging friction energy from rolling tires. Nano Energy.

[16] Kim M.K., Kim M.S., Jo S.E., Kim Y.J. (2016). Triboelectric–thermoelectric hybrid nanogenerator for harvesting frictional energy. Smart Mater. Struct..

[17] Ouyang H., Tian J., Sun G., Zou Y., Liu Z., Li H., Zhao L., Shi B., Fan Y., Fan Y. (2017). Self-powered pulse sensor for antidiastole of cardiovascular disease. Adv. Mater..

[18] Xu S., Zhang Y., Cho J., Lee J., Huang X., Jia L., Fan J.A., Su Y., Su J., Zhang H. (2013). Stretchable batteries with self-similar serpentine interconnects and integrated wireless recharging systems. Nat. Commun..

[19] Weng W., Sun Q., Zhang Y., He S., Wu Q., Deng J., Fang X., Guan G., Ren J., Peng H. (2015). A gum-like lithium-ion battery based on a novel arched structure. Adv. Mater..

[20] Gaikwad A.M., Zamarayeva A.M., Rousseau J., Chu H., Derin I., Steingart D.A. (2012). Highly stretchable alkaline batteries based on an embedded conductive fabric. Adv. Mater..

[21] Yan C., Wang X., Cui M., Wang J., Kang W., Foo C.Y., Lee P.S. (2014). Stretchable silver-zinc batteries based on embedded nanowire elastic conductors. Adv. Energy Mater..

[22] Jia W., Wang X., Imani S., Bandodkar A.J., Ramiírez J., Mercier P.P., Wang J. (2014). Wearable textile biofuel cells for powering electronics. J. Mater. Chem. A.

[23] Halamkova L., Halamek J., Bocharova V., Szczupak A., Alfonta L., Katz E. (2012). Implanted Biofuel Cell Operating in a Living Snail. J. Am. Chem. Soc..

[24] MacVittie K., Halamek J., Halamkova L., Southcott M., Jemison W.D., Lobeld R., Katz E. (2013). From “cyborg” lobsters to a pacemaker powered by implantable biofuel cells. Energy Environ. Sci..

[25] Zebda A., Gondran C., le Goff A., Holzinger M., Cinquin P., Cosnier S. (2011). Mediatorless high-power glucose biofuel cells based on compressed carbon nanotube-enzyme electrodes. Nat. Commun..

[26] Chang H.-K., Choi E., Park J. (2016). Paper-based energy harvesting from salinity gradients. Lab Chip.

[27] Pang S., Gao Y., Choi S. (2017). Flexible and stretchable biobatteries: Monolithic integration of membrane-free microbial fuel cells in a single textile layer. Adv. Energy Mater..

[28] Desmet C., Marquette C.A., Blum L.J., Doumèche B. (2016). Paper electrodes for bioelectrochemistry: Biosensors and biofuel cells. Biosens. Bioelectron..

[29] Mohammadifar M., Choi S. (2017). A Papertronic, On-Demand and Disposable Biobattery: Saliva-Activated Electricity Generation from Lyophilized Exoelectrogens Preinoculated on Paper. Adv. Mater. Technol..

[30] Kwon C.H., Ko Y., Shin D., Kwon M., Park J., Bae W.K., Lee S.W., Cho J. (2018). High-power hybrid biofuel cells using layer-by-layer assembled glucose oxidase-coated metallic cotton fibers. Nat. Commun..

[31] Zhang L., Zhou M., Wen D., Bai L., Lou B., Dong S. (2012). Small-size biofuel cell on paper. Biosens. Bioelectron..

[32] Fraiwan A., Mukherjee S., Sundermier S., Lee H.-S., Choi S. (2013). A paper-based microbial fuel cell: Instant battery for disposable diagnostic devices. Biosens. Bioelectron..

[33] Gonzalez-Guerrero M.J., Del Campo F.J., Esquivel J.P., Leech D., Sabate N. (2017). Paper-based microfluidic biofuel cell operating under glucose concentrations within physiological range. Biosens. Bioelectron..

[34] Rewatkar P., Goel S. (2018). Paper-Based Membraneless Co-Laminar Microfluidic Glucose Biofuel Cell with MWCNT-Fed Bucky Paper Bioelectrodes. IEEE Trans. Nanobiosci..

[35] Villarrubia C.W.N., Lau C., Ciniciato G.P., Garcia S.O., Sibbett S.S., Petsev D.N., Babanova S., Gupta G., Atanassov P. (2014). Practical electricity generation from a paper based biofuel cell powered by glucose in ubiquitous liquids. Electrochem. Commun..

[36] Villarrubia C.W.N., Soavi F., Santoro C., Arbizzani C., Serov A., Rojas-Carbonell S., Gupta G., Atanassov P. (2016). Self-feeding paper based biofuel cell/self-powered hybrid μ-supercapacitor integrated system. Biosens. Bioelectron..

[37] Seok S., Lefeuvre E., Wong C., Park J. (2019). Electrical Characterization of Textile-Based Enzymatic Biofuel Cell for Energy Harvesting Interface Circuit. Proceedings of the 2019 Symposium on Design, Test, Integration & Packaging of MEMS and MOEMS (DTIP).

[38] Ottman G.K., Hofmann H.F., Bhatt A.C., Lesieutre G.A. (2002). Adaptive Piezoelectric Energy Harvesting Circuit for Wireless Remote Power Supply. IEEE Trans. Power Electron..

[39] Ramadass Y.K., Chandrakasan A.P. (2010). An Efficient Piezoelectric Energy Harvesting Interface Circuit Using a Bias-Flip Rectifier and Shared Inductor. IEEE J. Solid State Circuts.

[40] Lefeuvre E., Badel A., Brenes A., Seok S., Yoo C.S. (2017). Power and frequency bandwidth improvement of piezoelectric energy harvesting devices using phase-shifted SECE interface circuit. J. Intell. Mater. Syst. Struct..

[41] Zhang X., Ren H., Pyo S., Lee J.-I., Kim J., Chae J. (2015). A High-Efficiency DC–DC Boost Converter for a Miniaturized Microbial Fuel Cell. IEEE Trans. Power Electron..

[42] Dallago E., Barnabei A.L., Liberale A., Torelli G., Venchi G. (2016). A 300-mV Low-Power Management System for Energy Harvesting Applications. IEEE Trans. Power Electron..

[43] Macrelli E., Romani A., Paganelli R.P., Camarda A., Tartagni M. (2015). Design of Low-Voltage Integrated Step-up Oscillators with Microtransformers for Energy Harvesting Applications. IEEE Trans. Circuits Syst. I Regul. Pap..

[44] Park J.D., Ren Z. Efficient Energy Harvester for Microbial Fuel Cells using DC/DC Converters. Proceedings of the IEEE Energy Conversion Congress and Exposition.

[45] Wang C., Shim E., Chang H.K., Lee N., Kim H.R., Park J. (2020). Sustainable and high-power wearable glucose biofuel cell using long-term and high-speed flow in sportswear fabrics. Biosens. Bioelectron..

[46] Milton R.D., Giroud F., Thumser A.E., Minteer S.D., Slade R.C. (2013). Hydrogen peroxide produced by glucose oxidase affects the performance of laccase cathodes in glucose/oxygen fuel cells: FAD-dependent glucose dehydrogenase as a replacement. Phys. Chem. Chem. Phys..

[47] Yu Y., Chen Z., He S., Zhang B., Li X., Yao M. (2014). Direct electron transfer of glucose oxidase and biosensing for glucose based on PDDA-capped gold nanoparticle modified graphene/multi-walled carbon nanotubes electrode. Biosens. Bioelectron..

[48] Ramanavicius A., Kausaite-Minkstimiene A., Morkvenaite-Vilkonciene I., Genys P., Mikhailova R., Semashko T., Voronovic J., Ramanaviciene A. (2015). Biofuel cell based on glucose oxidase from Penicillium funiculosum 46.1 and horseradish peroxidase. Chem. Eng. J..

[49] Xie X., Ye M., Liu C., Hsu P.-C., Criddle C.S., Cui Y. (2015). Use of low cost and easily regenerated Prussian Blue cathodes for efficient electrical energy recovery in a microbial battery. Energy Environ. Sci..

[50] Araminaitė R., Garjonytė R., Malinauskas A. (2012). Rotating disk electrode study of Prussian blue-and glucose oxidase-based bioelectrode. J. Electroanal. Chem..

[51] Liu S., Ju H. (2003). Reagentless glucose biosensor based on direct electron transfer of glucose oxidase immobilized on colloidal gold modified carbon paste electrode. Biosens. Bioelectron..

[52] Yazdi A.A., Preite R., Milton R.D., Hickey D.P., Minteer S.D., Xu J. (2017). Rechargeable membraneless glucose biobattery: Towards solid-state cathodes for implantable enzymatic devices. J. Power Sources.

[53] Zohdy M., El-Naggar A., Abdallah W. (1997). Silk screen printing of some reactive dyes on gamma irradiated wool fabrics. Polym. Degrad. Stab..

