# Supplementary material for: Autonomous Energy Harvester Based on Textile-Based Enzymatic Biofuel Cell for On-Demand Usage
Source: Sensors (Basel). 2020 Sep 3;20(17):5009. doi: 10.3390/s20175009 (PMC7506770; doi:10.3390/s20175009)
Supplement: Supplementary file 1 [file sensors-20-05009-s001.pdf]

sensors

MDPI

Supplementary Information

Autonomous Energy Harvester Based on Textile-Based Enzymatic Biofuel Cell for On-Demand Usage †

Seonho Seok [1,*], Cong Wang [2], Elie Lefeuvre [1] and Jungyul Park [2,*]

1 Center for Nanoscience and Nanotechnology (C2N), University-Paris-Saclay, 91120 Palaiseau, France; elie.lefeuvre@universite-paris-saclay.fr
2 Department of Mechanical Engineering, Sogang University, Mapo-gu, 04107 Seoul, Korea; wang.tsung@gmail.com
* Correspondence: seonho.seok@u-psud.fr (S.S.); sortpark@sogang.ac.kr (J.P.)

† This paper is an extended version of our paper published in: Seok, S.; Lefeuvre, E.; Wang, C.; Park, J. Electrical Characterization of Textile-Based Enzymatic Biofuel Cell for Energy Harvesting Interface Circuit. In Proceedings of 2019 Symposium on Design, Test, Integration & Packaging of MEMS and MOEMS (DTIP), Paris, France.

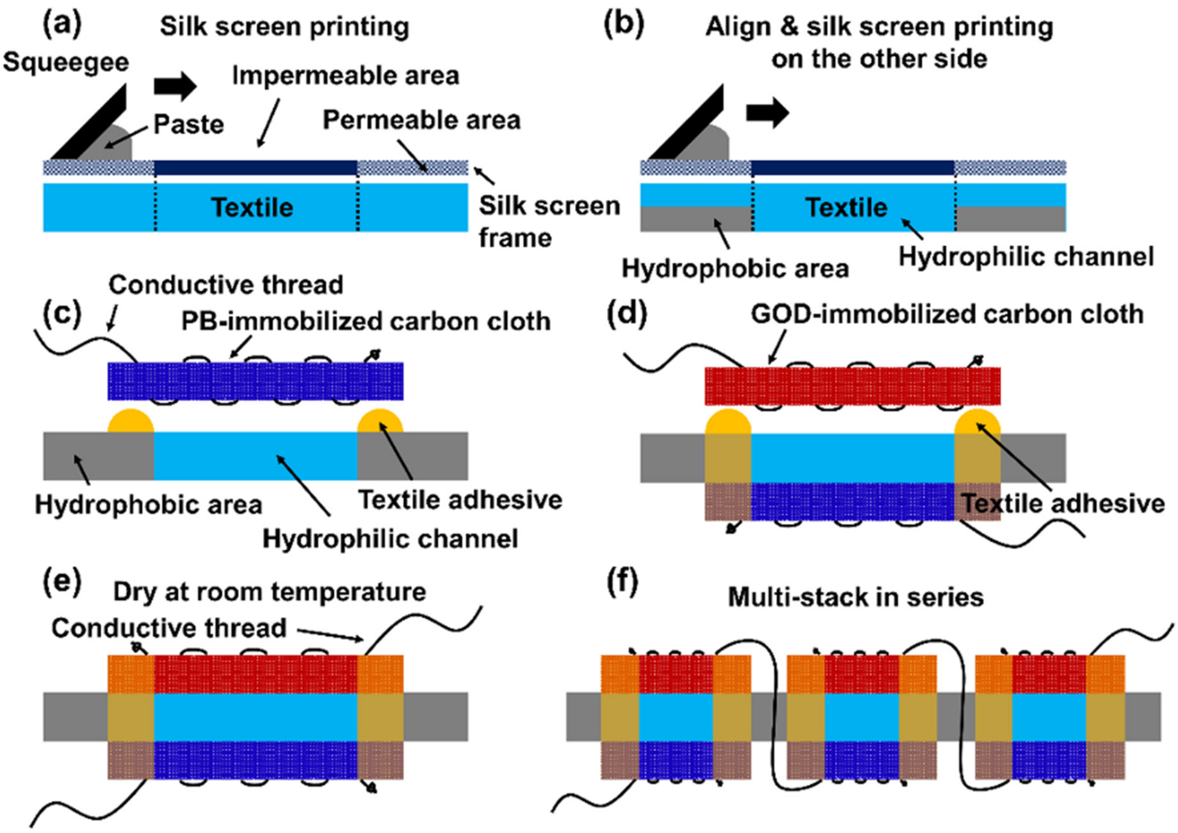

Figure S1. Fabrication process of the textile-based enzymatic biofuel cell. The biofuel cell consists of the transport layer, GOD-immobilized anode, PB-immobilized cathode, and conductive thread. The fabric was coated with solvent-based gutta serti N (Dupont, USA) using a silk screen printing procedure for hydrophobic coating (**a**). After printing on one side, the fabric was left to dry at room temperature. Then, the other side of the fabric was aligned with the silk screen frame and printed, following the same procedure, to ensure the hydrophobic coating completely permeated the fabric (**b**). Before attaching the carbon fabrics-based electrodes onto the transport layer, a stainless steel 2-ply conductive thread (Adafruit 640, New York City, NY, USA) was hand-sewn onto the carbon fabric

with a needle (**c**). It is used for collecting current and connecting the cell to external loads. The total width of the carbon fabric for the cathodes and anodes was 20 mm with a 5-mm margin on each side as bonding region, and the length was 10 mm and 30 mm for a one and three-stack fuel cell, respectively, in parallel. Thus, in the case of parallel connections, the effective working area of the electrodes was 10 × 10 mm^2 (one stack) and 10 × 30 mm^2 (three stacks) at the center. The two pieces of carbon fabric with the cathode and anode were bonded to the top and the bottom side of the transport layer, respectively, with solvent-free fabric adhesive (UHU textile), making sure the effective working area of the electrodes overlapped with the channel region (**c**–**d**). After the textile adhesive had fully dried, the preparation of a single fuel cell was completed (**e**). In addition, in the case of serial connections, fuel cell stacks were realized by connecting the cathode of one single fuel cell to the anode of another, using the conductive thread (**f**).

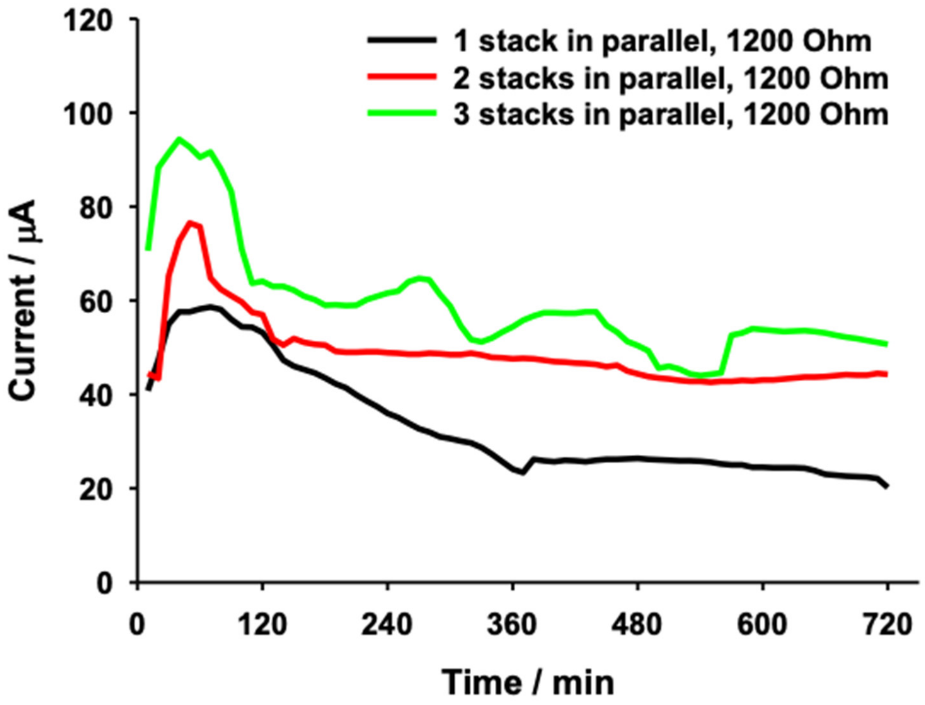

Figure S2. Long-term output of the biofuel cells when connected with an external resistance (1200 Ω).

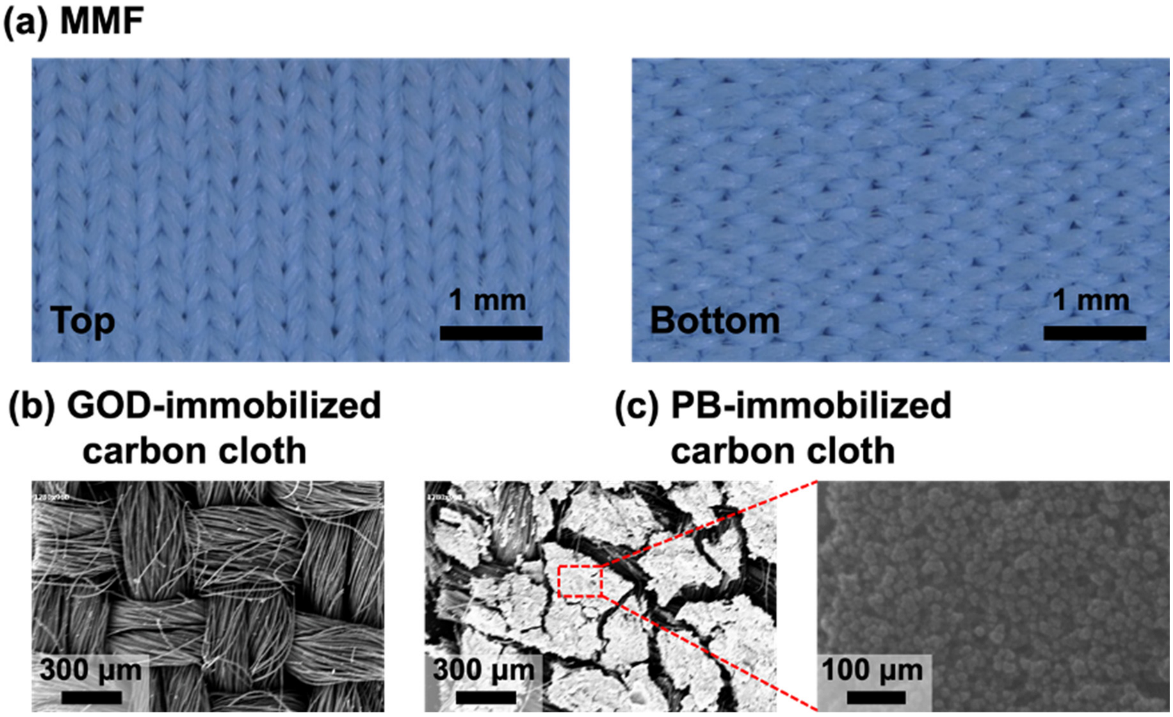

Figure S3. (**a**) The microscope image of MMF and the SEM images of (**b**) the GOD- and (**c**) PB-immobilized carbon cloth.

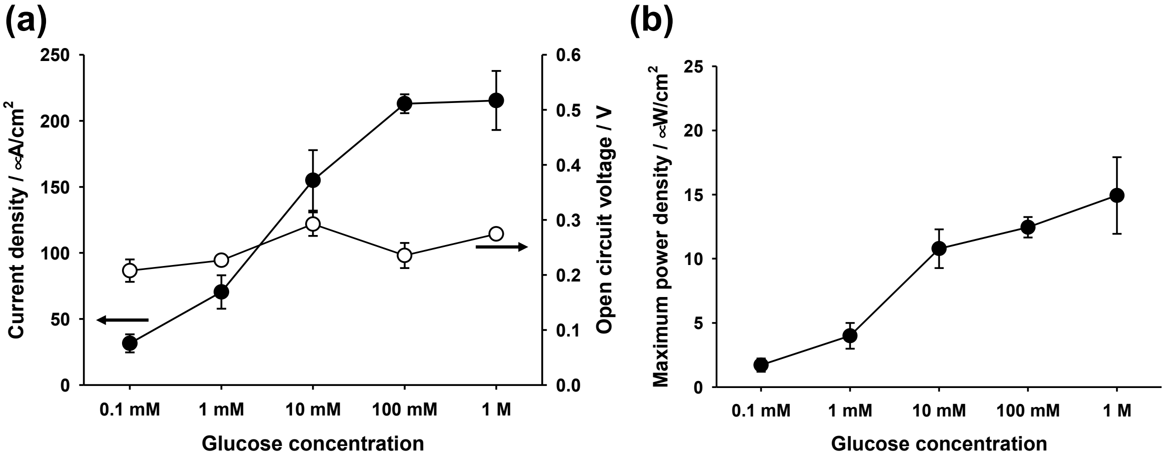

Figure S4. Variation of power generation according to the glucose concentration (**a**) The current density and (**b**) the maximum power density.
